# Liver resection by Ultrasonic Dissection and lntraoperative Ultrasonography

**DOI:** 10.1155/1996/98742

**Published:** 1996

**Authors:** Sherif S. Hanna, Robert Nam, Charlene Leonhardt

**Affiliations:** 1 Division of General Surgery Department of Surgery Sunnybrook Health Science Centre University of Toronto Toronto Ontario Canada; 2 Department of Radiology Sunnybrook Health Science Centre University of Toronto Toronto Ontario Canada

## Abstract

Ultrasonic dissetion (USD) and intraoperative ultrasonography (IOUS) have shown encouraging results in a retrospective analysis of 109 patients with benign or malignant liver disease. Of 109 patients assessed between 1980 and 1993, 84 were resected: 27 by finger fracture technique (FFT) and 57 by USD. Hospital mortality was 4.8% (4/84) and 30-day mortality was 6.0% (5/84). Overall morbidity was 48.8% (41/84) and liver related morbidity (hepatic bleeding, sepsis, and bile leak) was 34.5% (29/84); of the 29 patients, 5 required re-operation. Liver complications occurred in 12/27 (44.4%) in the FFT group as opposed to 17/57 (29.8%) in the USD group. The incidence of postoperative hepatic bleeding was significantly less by USD than by FFT(*p*=O.03). As well, intraoperative blood loss (*p*=O.01)number of intraoperative blood units used (*p*=0.002), and postoperative length of stay (*p*=O.O09) have been significantly reduced by USD. IOUS was used on 64 patients. Not only has it improved the sensitivity (99%) and specificity (98%) for detection of hepatic neoplasms, it has also helped increase the precision and accuracy of anatomical tumour localization. As a result, 11/64 patients (17.2%) had their preoperative plans changed: 8 were abandoned and 3 were revised. In summary, USD has significantly reduced intraoperative blood loss and hence reduced the number of intraoperative transfusions, incidence of postoperative complications and postoperative length of stay. IOUS should be routinely employed in patients undergoing liver resection since it provides critical information that could obviate oncologically useless resections.


*HPB Surgery*, 1996, Vol. 9, pp.121–128
Reprints available directly from the publisher
Photocopying permitted by license only

© 1996 OPA (Overseas Publishers Association)
Amsterdam B.V. Published in The Netherlands
by Harwood Academic Publishers GmbH
Printed in Malaysia

### Liver resection by Ultrasonic Dissection and Intraoperative Ultrasonography

*SHERIF S. HANNA, ROBERT NAM* and CHARLENE LEONHARDT+

*Division of General Surgery, Department of Surgery, Sunnybrook Health Science Centre, University of Toronto, Toronto, Ontario, Canada
+Department of Radiology, Sunnybrook Health Science Centre, University of Toronto, Toronto, Ontario, Canada

*(Received 20 July 1994)*

Ultrasonic dissetion (USD) and intraoperative ultrasonography (IOUS) have shown encouraging results in a retrospective analysis of 109 patients with benign or malignant liver disease. Of 109 patients assessed between 1980 and 1993, 84 were resected: 27 by finger fracture technique (FFT) and 57 by USD. Hospital mortality was 4.8% (4/84) and 30-day mortality was 6.0% (5/84). Overall morbidity was 48.8% (41/84) and liver related morbidity (hepatic bleeding, sepsis, and bile leak) was 34.5% (29/84); of the 29 patients, 5 required re-operation. Liver complications occurred in 12/27 (44.4%) in the FFT group as opposed to 17/57 (29.8%) in the USD group. The incidence of postoperative hepatic bleeding was significantly less by USD than by FFT($p=0.03$). As well, intraoperative blood loss ($p=0.01$) number of intraoperative blood units used ($p= 0.002$), and postoperative length of stay ($p=0.009$) have been significantly reduced by USD. IOUS was used on 64 patients. Not only has it improved the sensitivity (99%) and specificity (98%) for detection of hepatic neoplasms, it has also helped increase the precision and accuracy of anatomical tumour localization. As a result, 11/64 patients (17.2%) had their preoperative plans changed: 8 were abandoned and 3 were revised. In summary, USD has significantly reduced intraoperative blood loss and hence reduced the number of intraoperative transfusions, incidence of postoperative complications and postoperative length of stay. IOUS should be routinely employed in patients undergoing liver resection since it provides critical information that could obviate oncologically useless resections.

KEY WORDS: Liver resection ultrasonic dissection intraoperative ultrasonography

### INTRODUCTION

Since the first successful liver resections performed in the late 1800's, the evolution in surgical technique for hepatic resection has focused mainly on maximizing hemostasis.[1] Recently, ultrasonic dissection has been given considerable attention partly because of its widespread surgical applications[2,3,4]. As well, intraoperative ultrasonography has been able to give higher resolution images for better tumour localization and anatomical definition over any other preoperative radiological device[5]. By directly examining the liver with an ultrasound probe, it has allowed the surgeon to clearly map out each individuals' anatomical and pathological variances for precise and accurate resections.

The purpose of this study is to retrospectively assess how the ultrasonic dissector compares to the standard finger fracture technique with respect to hepatic related morbidity both intra-and postoperatively in major and minor liver resections. The influence of intraoperative ultrasonography on the decisions made during laparotomy is also examined.

### MATERIALS AND METHOD

Between 1980 and 1993, 109 patients were assessed for liver surgery by the primary author (S.S.H) at the Division of General Surgery, Sunnybrook Health Science Centre – University of Toronto (Toronto, Canada). Twenty and 89 patients presented with benign and malignant disease, respectively (Table 1). Their ages ranged from 21 to 78 years with a mean age of 56.2 fifty-one were female and 58 were male. Preoperative work-up included ultrasonography (US), computed tomography (CT) scan, and a serum

**Table 1** Patient distribution of presenting liver neoplasms

| Malignant Disease (n=89) | | No. |
|---|---|---|
| Metastases from: | colorectal cancer | 49 |
| | biliary/panc. ca. | 14 |
| | genitourinary ca. | 9 |
| | other ca. | 17 |
| Hepatocellular carcinoma | | 8 |
| Benign Disease (n=20) | | |
| Focal Nodular Hyperplasia | | 8 |
| Hemangioma | | 4 |
| Other | | 8 |

**Table 2** Types of resections performed (n=84)

| Major Resections (n=51) | USD | FFT |
|---|---|---|
| left lobectomies | 7 | 5 |
| right lobectomies | 10 | 8 |
| step left lobectomies* | 1 | 0 |
| step right lobectomies** | 7 | 0 |
| left trisegmentectomies | 0 | 1 |
| right trisegmentectomies | 6 | 5 |
| resection of 4 segments | 1 | 0 |
| | 32 | 19 |
| **Minor Resections (n=33)** | | |
| resection of ≤ 2 segments | 24 | 7 |
| resection of 3 segments | 2 | 0 |
| | 26 | 7 |

* left lobectomy + resection of segment 5
** right lobectomy + resection of segment 4A or 4B

biochemical liver profile in all patients. Where warranted, angiography, radionuclide scanning and percutaneous biopsies were also done to aid in the diagnosis. CT portography (CTP) was performed on 30 patients since its advent in 1990 and magnetic resonance imaging (MRI) was incorporated as a diagnostic test since 1991

All 109 patients were surgically explored. The final decision as to whether or not to proceed with resection was made during laparotomy. All explorations consisted of general inspection and palpation of the abdomen including careful examination of the liver and, since October 1989[6], intraoperative ultrasound (IOUS) of the liver. Patients with malignant disease were considered unresectable if there was diffuse hepatic disease, if there was extensive extrahepatic disease, or if the patient had advanced cirrhosis. Of the 109 patients explored, 84 were amenable to resection. The finger fracture technique (FFT) was used on 27 patients and the ultrasonic dissector (Cavitron Ultrasonic Surgical Aspirator, CUSA[R], Valleylab, Pfizer Co) was used on 57 patients; since 1988, the CUSA was routinely used on almost every patient thereafter. Fifty-one patients underwent major liver resections (lobectomies and trisegmentectomies) and 33 underwent minor resections (removal of 3 or fewer separate segments as defined by Couinaud) (Table 2). All resections performed by the primary author were intended for cure.

Since its introduction to the author's practice in October 1989, IOUS was used on 64 of the 109 patients analyzed. From the 27 patients in the FFT group, 3 had IOUS; from the 57 patients in the CUSA group, 31 had IOUS. Twenty of the 25 patients that were not resected also had IOUS. IOUS of the liver was done with an Aloka SSD-630 (Omnium Medical Devices, Ontario) sonographic unit with both linear 7.5 MHz (556T-75) and 5 MHz (587T-5) transducers. Liver surfaces were exposed for optimal imaging and were systematically examined in perpendicular planes. Neoplasms were carefully localized in accordance with Couinaud's segmental anatomy and in relation to key vascular structures such as the vena cava, the portal vein and the hepatic vein.

All patient records were maintained on a computer database (Smart and Smart II Database Systems, Informix Software, Inc.) for retrospective analysis. Statistical comparisons were made between the FFT group and the CUSA group. To make the comparisons more statistically uniform, separate comparisons were made with patients only undergoing major resections (lobectomies and trisegmentectomies) in the two groups. Estimated blood loss, the number of perioperative blood units used, and the postoperative length of stay were compared using Wilcoxon Rank Sum Tests. The rates of postoperative hepatic complications in the two groups were compared using Fisher's Exact Test. Postoperative hepatic complications included bile leak, sepsis and hepatic bleeding.

The influence of IOUS was measured by how many operative plans it altered and by how many neoplastic lesions it detected. This number was compared to the actual number determined by pathology in those patients who were resected. All patients would proceed to surgery from pre-operative assessment and then would have IOUS. Its result would influence outcome in one of three ways: proceed with resection plan; alter resection plan; or abandon the resection. If surgery was abandoned because of unforeseen tumour burden, it would be confirmed either by tissue biopsy examined by frozen section or by obvious gross and palpable intraopative assessment. A true positive was gauged by histological examination of each specimen. Five to 10 milimetre slices (depending on the size of the specimen) would be histologically examined for malignant cells after gross inspection. A true negative per se could not be measured since each specimen contained tumour.

The presence of false positives, however, which could be found from histological examination of all preoperative worked-up and IOUS detected lesions, requires some definition of a true negative to measure specificity. Thus, a true negative will be arbitrarily defined as the number of specimens in which the number of lesions found from either preoperative work-up or IOUS was equal to the number of lesions found by pathology.

### RESULTS

### *Mortality and Morbidity*

Overall hospital mortality (i.e. death within the same hospital admission) was 4.8% (4/84) and 30-day mortality was 6.0% (5/84). These included patients with advanced cirrhosis, severe jaundice and other premorbid factors (Table 3).

Overall morbidity was 48.8% (41/84) and hepatic related morbidity (hepatic bleeding, sepsis and bile leak) was 34.5% (29/84); 5/29 required re-operation (Table 4, Figure 1). All patients (7) who experienced hepatic bleeding alone underwent major resection; no patient experienced postoperative hepatic bleeding from minor resections. The patients that underwent major resections showed a hepatic related complication rate of 31.2% (10/32) in the USD group and 50.0% (9/18, 19th patient was intraoperative death) in the FFT group.

### *Operative Technique*

Table 5 and Figures 2–4 summarize the comparison between the USD and FFT group with respect to estimated blood loss (EBL), number of blood units (NBU) required and postoperative length of stay (PLOS).

**Table 3** Mortality analysis of resected patients

| Patient | Day of Death | Diagnosis | Complicating Features | Cause & Place of Death |
|---|---|---|---|---|
| 1 FFT | intraop | carcinoid syndrome | tricuspid regurgitation ventricular septal defect | hemorrhage in hosp. |
| 2 FFT | 5 POD* | renal cell ca. | – | MI**,PE+ in hosp |
| 3 FFT | 20 POD | bile duct ca. | severe jaundice | MI in hosp. |
| 4 USD | 25 POD | colorectal metastases | severe cardiac disease | MI at home |
| 5 USD | 30 POD | hepatoma | cirrhosis PBC++ | bleeding varices on re-adm. |
| 6 FFT | 36 POD | carcinoid syndrome | FFT-excessive IO hemorrhage | hemorrhage liver failure in hosp. |

* postoperative day
** myocardial infarction
\+ pulmonary embolus
\++ primary biliary cirrhosis

**Table 4** Morbidity analysis of resected patients

| Postoperative complication | Frequency | | |
|---|---|---|---|
| | USD (N=57) | FFT (n=27) | |
| non-hepatic related alone (wound infection, chest comp., jaundice, etc) | 14.0% | 14.8% | |
| hepatic related alone (hepatic bleeding, bile leak, sepsis) | 14.0% | 22.2% | |
| hepatic bleeding alone | 3.5% | 19.2% | p=0.03 |
| hepatic and non-hepatic related in combination | 15.7% | 22.2% | |
| either hepatic alone or with non-hepatic comb. | 29.8% | 44.4% | |

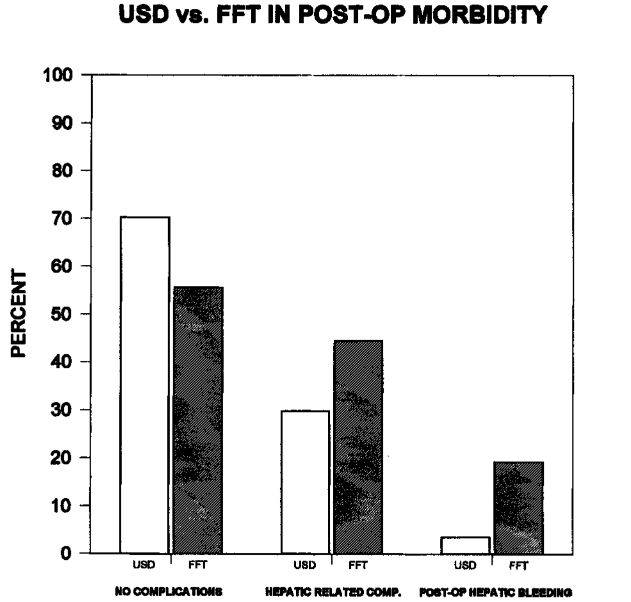

**Figure 1** Postoperative morbidity comparisons between ultrasonic dissection (USD) and finger fracture (FFT).

Of the 64 patients that underwent IOUS, 11 plans were altered (17.2%). Eight plans for resections were abandoned and 3 plans were amended. IOUS revealed 2 patients with diffuse unresectable hepatic metastases from colorectal cancer (Figure 5); 3 patients with lesions adherent to vascular structures (vena cava, the portal and hepatic veins) in which their nature made it technically unfeasible for resection (Figure 6); 1 with a tumour thrombus in the right portal vein from a hepatoma; 1 with a benign lesion from focal nodular hyperplasia that was left alone; and the eighth patient with a hepatoma and extensive cirrhosis whose resection was abandoned because IOUS detected more nodules which would have required an extended right lobectomy. Of the three remaining patients who had altered resections, IOUS revealed that one patient planned for a right lobectomy from metastases from a Klatskin tumour had only segments 4B and 5 involved and therefore only those segments were removed. In another patient planned for a right lobectomy from colorectal metastases, IOUS showed that the tumour extended into segment 4A and thus a step resection of 4A with the right lobectomy was performed. In the third patient planned for left lateral sectorectomy (segments 2 and 3) from colorectal metastases, IOUS revealed that the tumour involved portions of the left medial lobe and therefore an anatomic left lobectomy was performed.

**Table 5** Comparison of USD and FFT in estimated blood loss (EBL), number of blood units used (NBU), and postoperative length of stay (PLOS)

| | All Resections | | |
|---|---|---|---|
| | USD (n=57) | FFT (n=27) | p-value |
| mean EBL | 3765 mL | 9478 mL | 0.01 |
| range EBL | 350-24000 mL | 500-34000 mL | |
| mean NBU | 5.6 units | 16.4 units | 0.002 |
| range NBU | 0-35 units | 0-51 units | |
| mean PLOS | 18.6 days | 27.8 days | 0.009 |
| range PLOS | 5-76 days | 7-78 days | |
| | **Major Resections** | | |
| | USD (n=32) | FFT (n=19) | p-value |
| mean EBL | 4369 mL | 11641 mL | 0.0005 |
| range EBL | 350-21500 mL | 550-34000 mL | |
| mean NBU | 6.9 units | 20.4 units | 0.0002 |
| range NBU | 0-28 units | 0-51 units | |
| mean PLOS | 19.6 days | 35.4 days | 0.003 |
| range PLOS | 6-70 days | 12-78 days | |

### *Intraoperative Assessment*

Forty patients within the database had IOUS and records available revealing number of hepatic neoplasms found by pathology; a total of 68 liver nodules were found. Sensitivity and specificity rates are compared in Table 6. The single false positive from IOUS was a hemangioma lesion detected in the caudate lobe that could not be confirmed by pathology.

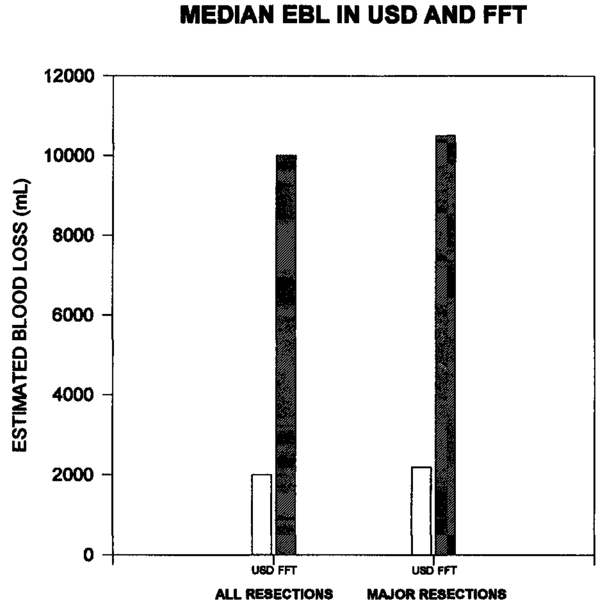

**Figure 2** Comparison of the median estimated blood loss (EBL) between ultrasonic dissection (USD) and finger fracture (FFT)

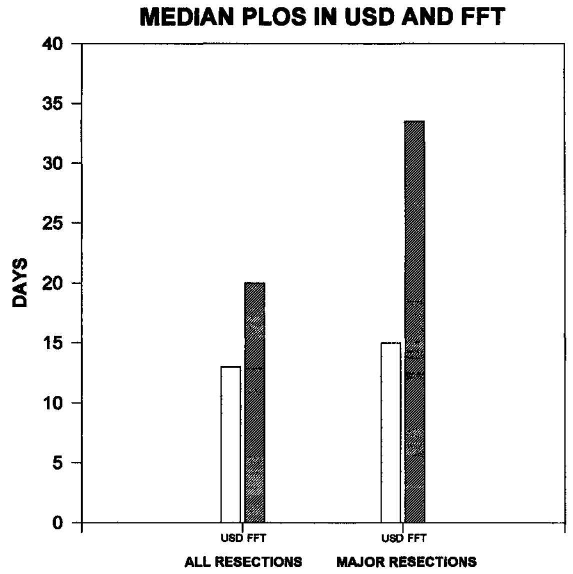

**Figure 4** Comparison of the median hospital postoperative length of stay (PLOS) between ultrasonic dissection (USD) and finger fracture (FFT).

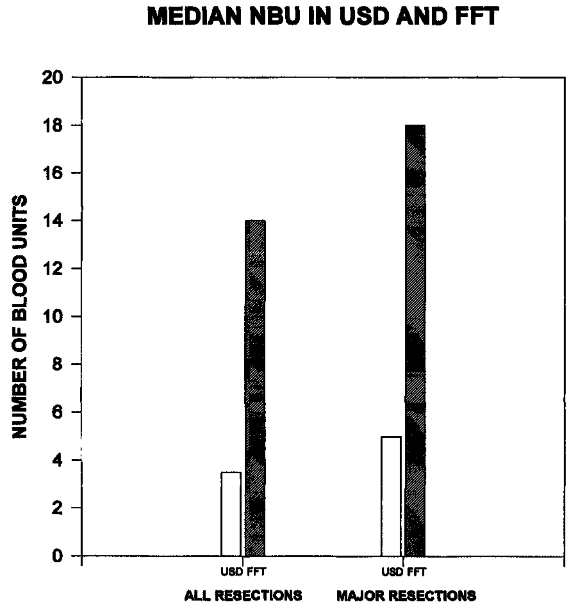

**Figure 3** Comparison of the median number of blood units used intraoperatively (NBU) between ultrasonic dissection (USD) and finger fracture (FFT).

**Table 6** IOUS assessment. TP = true positive; TN = true negative; FP = false positive, FN = false negative

| | TP | TN | TP | FN |
|---|---|---|---|---|
| Preoperative work-up* | 68 | 36 | 4 | 9 |
| IOUS assessment | 68 | 39 | 1 | 1 |
| | Sensitivity+ | | specificity++ | |
| Preoperative work-up* | 88% | | | 90% |
| IOUS assessment | 99% | | | 98% |

* includes pre-op ultrasonography, CT scan ±portography, ±MRI scan, ±radionuclide scanning.
\+ sensitivity = TP/(TP+FN)
++ specificity = TN/(TN+FP)

### DISCUSSION

### *Ultrasonic Dissection*

The principle behind ultrasonic dissection (USD) is based on the premise that different tissues have different water content in proportion to collagen and elastin density. Hepatic tissue with high water content can therefore be selectively fragmented at a given frequency leaving numerous collagen rich blood vessels and bile ducts intact for safe ligation.[7] Thus, blood loss is minimized and a cleaner resection line is obtained.

Hemorrhage is the most important intraoperative and postoperative complication in liver surgery.[8–11] Little *et al.* found that USD significantly reduced perioperative blood loss and the number of transfusions required.[12] Reports have shown that the higher the blood loss, the greater the incidence of postoperative complications.[8,10] Although the determinants for postoperative length of stay (PLOS) is multifactorial, the degree of intraoperative and postoperative blood loss plays a major role. Indeed, PLOS was, on average, approximately 10 days shorter in the USD group which reduced health care costs by 30% in our series.

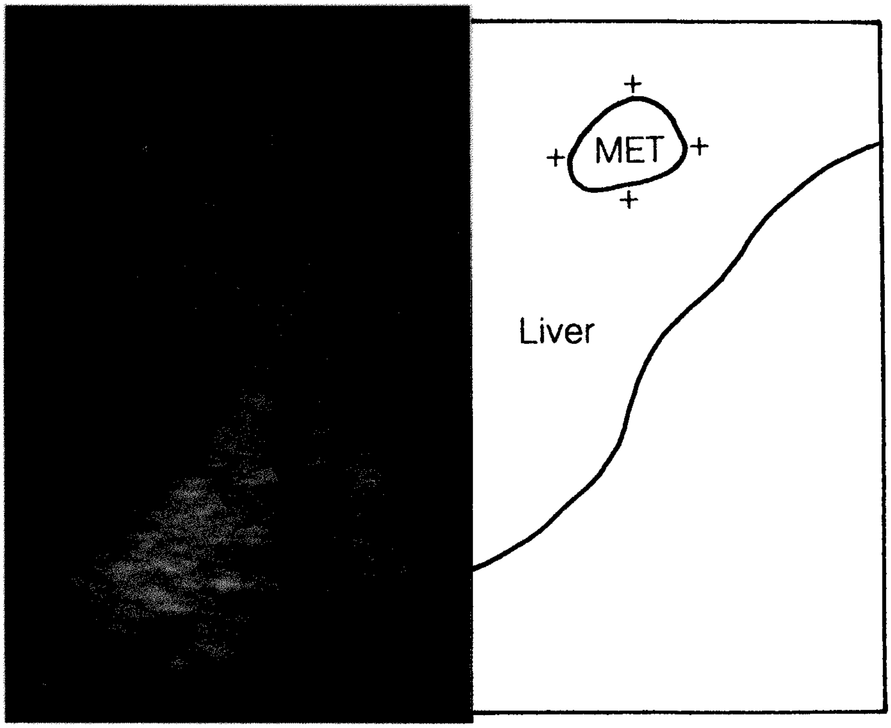

**Figure 5** IOUS detection of extra lesion (MET) that was not detected by preoperative computed tomography. Similar lesions were found elsewhere in the liver and resection was subsequently abandoned.

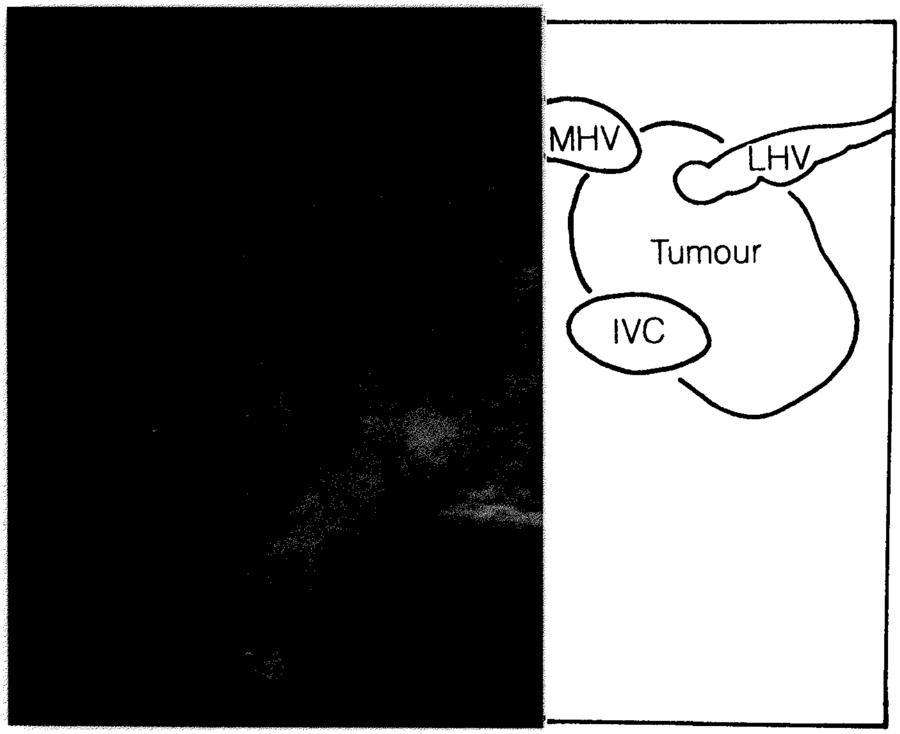

**Figure 6** IOUS detection of extra tumour involvement with the middle hepatic vein (MHV), left hepatic vein (LHV) and inferior vena cava (IVC) which were not discernable by preoperative computed tomography. Resection was subsequently abandoned.

**Table 7** Literature report of IOUS sensitivity and specificity

| | patients | sensitivity | specificity |
|---|---|---|---|
| Castaing *et al.*[25] | 42 | 78.5% | 100.0% |
| Gozzetti *et al.*[28] | 37 | 97.8% | – |
| Haider *et al.*[30] | 22 | 91.8% | 100.0% |
| Igawa *et al.*[31] | 83 | 90.9% | 96.0% |
| Machi *et al.*[32] | 84 | 97.8% | 94.0% |
| Machi *et al.*[5] | 227 | 93.3% | 94.7% |
| Makuuchi *et al.*[33] | 152 | 99.0% | – |
| Nagasue *et al.*[27] | 113 | 98.0% | – |
| Parker *et al.*[34] | 42 | 98.0% | – |
| Rifkin *et al.*[35] | 49 | 100.0% | 100.0% |
| Sheu *et al.*[26] | 47 | 96.5% | – |
| Hanna *et al*[**] | 64 | 99.0% | 98.0% |

* values apply only in detecting intrahepatic metastases.
** present report.

It has been further suggested that high perioperative blood loss requiring numerous blood transfusions may have an unfavourable prognostic influence on long-term survival in colorectal metastatic disease since blood loss may adversely influence immunocompetence.[13-15] Although the exact mechanism is unclear, experiments performed on tumour bearing mice and rats have clearly shown increased tumour growth as a result of blood transfusions possibly by affecting lymphocyte and macrophage activity.[16-19]

USD also decreases the amount of ischemic tissue at the resection margin in controlled animal experiments.[20,21] This may explain why the USD group experienced less morbidity since the degree of devitalized tissue after the resection may be directly related to the severity of sepsis which is a major contributor towards postoperative morbidity.[22-24]

It should be mentioned that subjectively, USD increased the time required for the liver transection phase compared to finger fracture, but once the liver transection was complete hemostasis was superior to finger fracture and this most likely resulted in no difference in overall operative time.

*Intraoperative Ultrasonography*

The advantage of scanning with an ultrasound probe directly across the liver surface has allowed for more accurate anatomic localization of neoplasms. Because there is no need to penetrate the abdominal wall, high frequency transducers (7.5 MHz) can be used to generate high resolution images that cannot be obtained preoperatively.[25] As a result, IOUS saved 8 patients from unnecessary resections that would have otherwise resulted in considerable morbidity and possible mortality. It altered the plans of 3 patients by changing the amount of resected liver to allow for greater tumour free resection margins. IOUS revealed vital information specifically entailing the location of tumours in relation to major vessel structures and segmental anatomy (Figure 5, 6).

As well, IOUS is particularly important in patients with hepatocellular carcinoma and underlying cirrhosis. Not only has IOUS been effective in detecting small hepatomas that could not be palpated during laparotomy or detected preoperatively,[26-28] but it has also been able to map out more conservative resections, and thereby maintain adequate hepatic reserve in cirrhotic livers.

Conversely, by detecting extra smaller nodules of hepatocellular carcinoma, IOUS may also avoid any unwarranted resections (as it did with one patient with cirrhosis in our series).

Despite careful preoperative imaging with CT-portography, ultrasound, and MRI, the sensitivity and specificity of IOUS have been higher. Other authors show similar results as summarized in Table 7. Despite some concerns such as increasing operative time and intra-interpreter variability[29], the overwhelming consensus in the high sensitivity and specificity rates reported internationally (Table 7) indicate the invaluable information that can be obtained from IOUS. It should therefore be regularly incorporated as the final diagnostic test prior to proceeding with liver resection.

### REFERENCES

1. Meyers, W.C. and Jones, R.S. (1990) *Textbook of Liver and Biliary Surgery*, pp. 391-399. Philadelphia: J.B. Lippincott Co.
2. Flamm, E.S., Ransohoff, J., Wunchinich, D. and Broadwin, A. (1978) Preliminary experience with ultrasonic aspirator in neurosurgery. *Neurosurgery*, 2, 240–245

3. Addonizio, J.C. and Choudhury, M.S. (1986) Cavitrons in urologic surgery. *Urologic Clinics of North America*, **13**, 445–454
4. Deppe, G., Vinay, K.M. and Malone, J.M. (1988) Debulking surgery for ovarian cancer with the Cavitron Ultrasonic Surgical Aspirator (CUSA)—A preliminary report. *Gynecologic Oncology*, **31**, 223–226
5. Machi, J., Sigel, B., Zaren, H.A., Kurohiji, T. and Yamashita, Y. (1993) Operative ultrasonography during hepatobiliary and pancreatic surgery. *World J. Surg.*, **17**, 640–646
6. Hanna, S.S., Withers, C., Arenson, A-M., Hamilton, P., Leonhardt, C. and Towers, M. (1992) Role of intraoperative ultrasonography in hepatic surgery: a preliminary report. *Can. J. Surg.*, **35**, 151–153
7. Hodgson, W.J.B., Poddar, P.K., Mencer, E.J., Williams, J., Drew, M. and McElhinney, A.J. (1979) Evaluation of ultrasonically powered instruments in the laboratory and in the clinical setting. *Am. J. Gastroenterol*, **72**, 133–140
8. Nagao, T., Inoue, S., Mizuta, T., Saito, H., Kawano, N. and Morioka, Y. (1985) One Hundred Hepatic Resections: Indications and operative results. *Ann. Surg.*, **202**, 42–49
9. Cole, D.J. and Ferguson, C.M. (1992) Complications of hepatic resection for colorectal carcinoma metastasis. *Am. Surgeon*, **58**, 88–91
10. Holm, A., Bradley, E. and Aldrete, J.S. (1989) Hepatic resection of metastasis from colorectal carcinoma: morbidity, mortality and pattern of recurrence. *Ann. Surg.*, **209**, 428–434
11. Ekberg, H., Tranberg, K.G., Andersson, R., Jeppsson, B. and Bengmark, S. (1986) Major liver resection: perioperative course and management. *Surgery*, **100**, 1–7
12. Little JM, Hollands MJ. (1991) Impact of the CUSA and operative ultrasound on hepatic resection. *HPB Surgery*, **3**, 271–278
13. Younes, R.N., Rogatko, A. and Brennan, M.F. (1991) The influence of intraoperative hypotension and perioperative blood transfusion on disease-free survival in patients with complete resection of colorectal lover metastases. *Ann. Surg.*, **214**, 107–113
14. Van Ooijen, B., Wiggers, T., Meijer, S., *et al.* (1992) Hepatic Resections for colorectal metastases in The Netherlands: A multiinstitutional 10-year study. *Cancer*, **70**, 28–34
15. Jubert, A.V., Lee, E.T., Hersh, E. and McBride, C.M. (1973) Effects of surgery, anesthesia and intraoperative blood loss on immunocompetence. *J. Surg. Res.*, **15**, 399–403
16. Younes, R.N., Rogatko, A., Vydelingum, N.A. and Brennan, M.F. (1991) Effects of hypovolemia and transfusion on tumor growth in MCA-tumor-bearing rats. *Surgery*, **109**, 307–312
17. Lieberman, M.D., Shou, J., Sigal, R.K., Yu, J., Goldfine, J. and Daly, J.M. (1990) Transfusion-induced immunosuppression results in diminished host survival in a murine neuroblastoma model. *J. Surg. Research*, **48**, 498–503
18. Wu, H-S. and Little, A.G. (1988) Perioperative blood transfusions and cancer recurrence. *J. Clinical Oncology*, **6**, 1348–1354
19. Giacchino, F., Coppo, R., Giachino, G., Belardi, P., Pellerey, M. and Piccoli, G. (1982) The influence of blood transfusion on cellular immunity. *Immonology Letters*, **5**, 253–257
20. Ottow, R.T., Barbieri, S.A., Sugarbaker, P.H. and Wesley, R.A. (1985) Liver transection: a controlled study of four different techniques in pigs. *Surgery*, **97**, 596–601
21. Tranberg, K-G., Rigotti, P., Brackett, K.A., Bjornson, H.S., Fischer, J.E. and Joffe, S.N. (1986) Liver Resection: a comparison using the Nd-YAG laser, an ultrasonic surgical aspirator, or blunt dissection. *Am. J. Surg*, **151**, 368–373
22. Pace, R.F., Blenkharn, J.I., Edwards, W.J., Orloff, M., Blumgart, L.H. and Benjamin, I.S. (1989) Intra-abdominal sepsis after hepatic resection. *Ann. Surg.*, **209**, 302–306
23. Wang, X., Andersson, R., Soltesz, V. and Bengmark, S. (1992) Bacterial translocation after major hepatectomy in patients and rats. *Arch. Surg.*, **127**, 1101–1106
24. Fortner, J.G., Kim, D.K., Maclean, B.J., *et al.* (1978) Major hepatic resection for neoplasia: personal experience in 108 patients. *Ann. Surg.*, **209**, 302–306
25. Castaing, D., Emond, J., Bismuth, H. and Kunstlinger, F. (1986) Utility of operative ultrasound in the surgical management of liver tumors. *Ann. Surg.*, **204**, 600–605
26. Sheu, J.C., Lee, C.S., Sung, J.L., Chen, D.S., Yang, P.M. and Lin, T.Y. (1985) Intraoperative hepatic ultrasonography—an indispensable procedure in resection of small hepatocellular carcinomas. *Surgery*, **97**., 97–103
27. Nagasue, N., Kohno, H., Chang, Y.C., *et al.* (1989) Intraoperative ultrasonography in resection of small hepatocellular carcinoma associated with cirrhosis. *Am. J. Surg.*, **158**, 40–42
28. Gozzetti, G., Mazziotti, A., Bolondi, L., *et al.* (1986) Intraoperative ultrasonography in surgery for liver tumors. *Surgery*, **99**, 523–529
29. de Jong, K.P., Terpstra, O.T., Blankensteijn, J.D. and Lameris, J.S. (1989) Intraoperative ultrasonography and ultrasonic dissection in liver surgery. *Am. J. Gastroenterol.*, **84**, 933–936
30. Heider, M.A., Leonhardt, C., Hanna, S.S. and Tennenhouse, J. (1993) The role of intraoperative ultrasound in hepatic resections. Accepted for publication by *Journal of Radiology*
31. Igawa, S., Sakai, K., Kinoshita, H. and Hirohashi, K. (1985) Intraoperative sonography: clinical usefulness in liver surgery. *Radiology*, **156**, 473–478
32. Machi, J., Isomoto, H., Yamashita, Y., Korohiji, T., Shirouzu, K. and Kakegawa, T. (1987) Intraoperative ultrasonography in screening for liver metastases from colorectal cancer: comparative accuracy with traditional procedures. *Surgery*, **101**, 678–684
33. Makuuchi, M., Hasegawa, H., Yamazaki, S., Takayasu, K. and Moriyama, N. (1987) The use of operative ultrasound as an aid to liver resection in patients with hepatocellular carcinoma. *World J. Surg.*, **11**, 615–621
34. Parker, G.A., Lawrence, W., Horsley, J.S., *et al.* (1989) Intraoperative ultrasound of the liver affects operative decision making. Ann. Surg., 209 569–577
35. Rifkin, M.D., Rasato, F.E., Branch, H.M. *et al.* (1987) Intraoperative ultrasound of the liver: an important adjuctive tool for decision making in the operative room. Ann. Surg., 205, 466–472

